# Sexual and Reproductive Health Services and Help-seeking Behaviors: Experiences and Perspectives of Young 1.5-Generation Bangladeshi Women in Toronto

**DOI:** 10.1177/08445621251356734

**Published:** 2025-07-08

**Authors:** Nahela Nowshin, Lydia Kapiriri, Colleen M. Davison

**Affiliations:** 1 Department of Health, Aging & Society, 3710 McMaster University, Hamilton, Ontario, Canada; 2 Department of Public Health Sciences, 4257 Queen's University, Kingston, Ontario, Canada

**Keywords:** sexual health services, 1.5 generation, Bangladeshi, qualitative, Canada

## Abstract

**Background:**

The “1.5 generation” refers to those who immigrated to a new country as children or adolescents. In the context of Canada, 1.5-generation Bangladeshis are underrepresented in the extant literature on sexual and reproductive health (SRH). Their cross-cultural positionality and ethnic background have implications for their SRH-related help-seeking behaviors and service utilization.

**Purpose:**

This paper explored the SRH related help-seeking behaviors and perspectives and experiences with accessing and utilizing SRH services among 1.5-generation Bangladeshi women in Toronto, Ontario.

**Methods:**

Ten 1.5-generation Bangladeshi women, aged between 18 and 22, participated in this qualitative study. This study employed a narrative inquiry methodological approach and the Conceptual Framework of Access to Healthcare as a theoretical lens.

**Results:**

Knowledge about routine SRH tests and utilization of these services were found to be low. Mothers, sisters, peers, the internet and mobile apps were identified as sources of informal help-seeking. The family doctor is the most significant source of formal help-seeking. The findings shed light on the demand- and supply-side dimensions of access to SRH care as well as the multiple barriers and facilitators to services. Individual-level social and cultural factors in SRH help- and care-seeking behaviors and attitudes were identified. Structural factors within the health system hindered effective service delivery.

**Conclusion:**

The findings highlight the need for SRH-focused educational and informational campaigns in residential neighborhoods concentrated by the Bangladeshi diaspora. Policymakers should address the structural barriers in the health system to improve the quality of SRH care. Further investigation is required to understand what culturally responsive SRH care entails for 1.5-generation patients from South Asian and Muslim backgrounds.

## Introduction

The term “1.5-generation” refers to individuals who immigrated to a new country as children or early adolescents. Although the literature is ambiguous on what age children should immigrate to belong to the 1.5 generation (Li, 2021), there is a consensus that the 1.5ers are not identical to either the first or second generation because they are better adapted to the host country than their parents who immigrated as adults and have some degree of socialization in the country of origin unlike the second generation (Li, 2021; Liu, 2017; Roh & Chang, 2020). The cross-cultural positionality of the 1.5 generation—acculturation to the host society and internalization of the norms and values of the culture of origin—has implications for their constructions of sexual health and wellbeing and service- and help-seeking behaviors (Dune & Mapedzahama, 2017; Dune et al., 2017, 2021). The planning and design of sexual and reproductive health (SRH) services for the 1.5 generation should take into consideration a possible culture clash arising from the conflicting messages about sexual health received from their parents (whose constructions of SRH may be rooted in the culture of origin) and in school and the media (Dune & Mapedzahama, 2017).

Help-seeking entails communicating with others to acquire information, assistance, or general support when faced with a problem or stressful experience (Gourash, 1978; Rickwood et al., 2005). It may take the form of acquiring support from friends, relatives, and parents (informal) or seeking professional help through social and health institutions (formal) (Barker et al., 2005; Gourash, 1978). Informal help-seeking is often the first step in addressing SRH concerns (Åkerman et al., 2017; Moreira et al., 2005). Perceived social support has been found to facilitate informal and formal help-seeking (Hedge et al., 2017). When family members or friends are available to offer support, it can boost informal help-seeking intentions (asking family or friends for support) which, in turn, leads to increased formal help-seeking intentions (Hedge et al., 2017). Culture, family, intimate partners, and peers can shape perceptions toward formal help-seeking and decision-making about availing professional help (Aloud & Rathur, 2009; Cusack et al., 2004; Hedge et al., 2017; Wu et al., 2012).

A study conducted by Dune et al. (2021) sheds some light on the role of cultural connectedness and SRH-related help-seeking. The authors assessed cultural connectedness based on how strongly 1.5-generation migrants in Australia identified with the culture of their country of origin and with Australian culture. They observed that connectedness with both cultures facilitated formal and informal help-seeking, suggesting that identification with the culture of origin does not necessarily inhibit formal help-seeking among 1.5-generation migrants. The concept of cultural connectedness has more prominently been applied in research on Indigenous populations and health outcomes (Cueva et al., 2020; Snowshoe et al., 2015, 2017). Cultural connectedness is a multidimensional concept that entails commitment to one's culture, knowledge of and engagement with the traditional practices and language, and connection to aspects of spirituality (Snowshoe et al., 2015, 2017).

Although the literature on immigrant populations’ SRH behaviors and service barriers is vast, empirical studies at the intersection of SRH and migrants belonging to the 1.5-generation are limited (Dune et al., 2017). In Australia and the United States, 1.5-generation migrants reported costs, not knowing where to access care, concerns about confidentiality, lack of health insurance, lack of proficiency in English, and discrimination by health providers as some of the barriers to SRH care (Dune et al., 2021; Morales-Alemán et al., 2020). Luu et al. (2020) reported lack of knowledge about cervical cancer, not being recommended by a provider, low perceived risk, lack of health coverage, and stigma related to SRH as barriers to cervical cancer screening among 1.5- and second-generation Vietnamese-American women.

Empirical evidence on the help- and service-seeking attitudes of under-researched ethnic minorities and immigrant groups would be useful for primary care and public health practitioners whose contribution is necessary to meet Canada's national targets related to population SRH. Canada's targets include lowering the incidence of sexually transmitted and blood-borne infections (STBBIs), reducing stigma and discrimination that give rise to vulnerabilities to STBBIs, and improving access to testing and treatment (Centre for Communicable Diseases and Infection Control, 2018). Outlined in Canada's STBBI action plan 2024–2030 are key populations disproportionately impacted by STBBIs due to social and structural inequities (e.g., First Nations, Inuit, and Métis communities, immigrants, women and youth). These targets are aligned with the equity-driven approach toward controlling sexually transmitted infections (STIs) in the 2030 Agenda for Sustainable Development. Sustainable Development Goals 3 and 5 and their corresponding targets highlight the need to ensure universal access to SRH services, reproductive rights of women, and gender equality (Chersich et al., 2018).

South Asian girls and women in Canada experience numerous barriers to SRH care. These include low levels of knowledge about cervical screening, lack of knowledge about the Canadian health care system, fear of being seen accessing SRH services by family/community members, language barriers, and discrimination in health settings (Devotta et al., 2024; Gupta et al., 2002; Habib, 2012; Meherali et al., 2021). Cervical screening knowledge and testing behaviors among South Asian women were found to be dependent on multiple intersecting factors such as age/generation, formal education, rural/urban setting of place of emigration, level of acculturation and English proficiency (Gupta et al., 2002; Habib, 2012). In the extant literature on access to SRH care among South Asians in Canada, Bangladeshi women have been grouped together with other South Asian ethnic groups (e.g., Indians, Pakistanis) and 1.5-generation women have not been explicitly identified or focused on in these studies (Habib, 2012; Machado et al., 2022; Meherali et al., 2021). To date, there has been limited focus on 1.5-generation Bangladeshi women's experiences with SRH services and help-seeking behaviors. This paper seeks to address this research gap by exploring the SRH-related help-seeking behaviors and perspectives and experiences with accessing and utilizing SRH services^1^ among 1.5-generation Bangladeshi women in Toronto, Ontario.

## Methods

### Study Design

This paper is part of the first author's doctoral research study on the SRH of 1.5-generation Bangladeshi women. Using a narrative inquiry methodological approach, this study aimed to collect rich life stories of young 1.5-generation Bangladeshi women and obtain insight into their SRH-related help-seeking behaviors and experiences and perspectives with accessing and utilizing services. Participants’ stories, recounted in their own words, provided a window into the meanings of the experiences from the perspective of the storyteller. These stories, which arise from a complex interaction of social influences and personal history (Clandinin & Rosiek, 2007), shed light on the multidimensionality of their help-seeking experiences. Narrative inquiry prioritizes the stories that individuals tell to make sense of their experiences (Connelly & Clandinin, 2006). Stories are the portal through which interpretations of the world are expressed (Connelly & Clandinin, 2006). The study of human experience through narrative inquiry enables exploration of the social, cultural and institutional contexts within which human experiences are situated (Clandinin & Rosiek, 2007).

### The Conceptual Framework of Access to Healthcare

**Figure 1.**
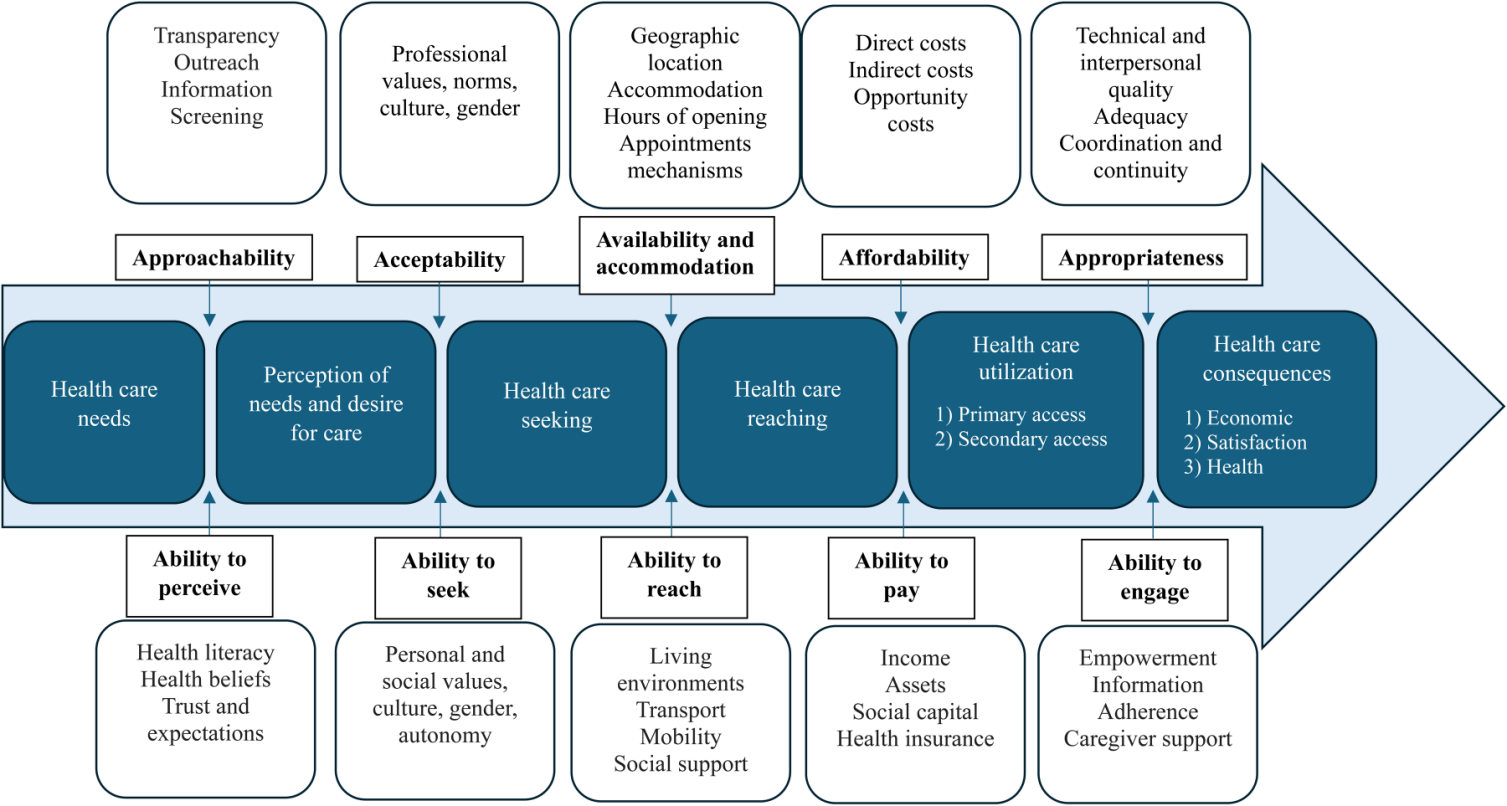
A Conceptual Framework of Access to Healthcare. Note: Reprinted from Levesque, J. F., Harris, M. F., & Russell, G. (2013). Patient-Centred Access to Health Care: Conceptualising Access at the Interface of Health Systems and Populations. International Journal for Equity in Health, 12, 18. https://doi.org/10.1186/1475-9276-12-18. Creative Commons (http://creativecommons.org/licenses/by/2.0).

Developed by Levesque et al. (2013), the Conceptual Framework of Access to Healthcare consists of a demand (health users) and supply side (health system), each containing five sequential dimensions. The supply side comprises five dimensions accompanied by corresponding dimensions on the demand side (Figure 1). Access is conceptualized as individuals’ ability to obtain appropriate care that meets their identified needs (Corscadden et al., 2017; Levesque et al., 2013). Levesque's framework not only takes into account the individual-level determinants that influence the ability to perceive, seek, reach, pay for, and engage in health care, but also the structural factors pertaining to the health system (e.g., transparency and provision of information by health providers, quality of treatment, professional values of physician). These dimensions are often interconnected (Levesque et al., 2013). For example, low health literacy would have an impact on an individual's understanding of a health problem (ability to perceive) as well as their capacity to communicate with their health provider and meaningfully participate in the care process (ability to engage). By considering both the health system and population contexts, the framework allows for the identification of barriers in accessing health care (Cu et al., 2021). Using Levesque's framework, previous research has explored barriers and facilitators to SRH services and assessed sexual health interventions (Hameed et al., 2020; Jozsa et al., 2023; Sidamo et al., 2024). In this study, Levesque's framework provided a critical lens to identify the barriers and facilitators of access to SRH care along the dimensions of the framework, i.e., the points of breakdown and/or continuity at different stages involved in the sequential process of obtaining SRH care.

### Recruitment and Participants

The inclusion criteria for participant selection were: (i) adult women of Bangladeshi origin; (ii) aged between 18 and 24; (iii) residing in Toronto, Ontario; (iv) born outside Canada; (v) immigrated to Canada as children or adolescents; and (vi) proficient in English. Purposive sampling was used to identify potential participants through the first author's personal network of acquaintances. The first author sought assistance from a Bangladeshi tutor based in Toronto to identify potential candidates. Social media posts were made in Facebook groups associated with the Bangladeshi diaspora in Canada. Due to the relative difficulties in identifying potential participants between the ages of 18–20 compared to older women, the snowball sampling technique was used to identify more participants in this age bracket. Ten 1.5-generation Bangladeshi women who grew up in Toronto, Ontario participated in this study. Their ages ranged from 18 to 22 and they immigrated to Canada between the ages of 6 and 13. All participants’ self-identified ethnic heritage is Bangladeshi. A summary of participants’ sociodemographic characteristics is provided in Table 1. All participants had the opportunity to read the study information sheet prior to signing the consent form. The information sheet was emailed to all participants. The sheet detailed the study objectives, procedures involved in the research, confidentiality and anonymity, and participation and withdrawal processes. All participants electronically signed the consent form prior to data collection.

**Table 1.** Summary of the Sociodemographic Characteristics of Participants.

Age group	*n* *=* *10*
18–20	4
21–22	6
Immigrated from	
Bangladesh	6
Botswana	1
Kuwait	1
Saudi Arabia	1
UAE	1
Age at immigration to Canada	
6–9	8
10–13	2
Marital status	
Married	2
Never married	8

### Data Collection and Analysis

Data were collected from December 2022 to August 2023 through in-depth semi-structured interviews. All interviews were conducted by the first author over the telephone in English and audio-recorded with permission. Participants received an honorarium of $30 (via online banking) for each interview session. A minimum of two interview sessions were carried out with each participant. Three interview sessions were conducted with participants who were unable to dedicate sufficient time for the first two sessions. More than one interview session was necessary with each participant given the breadth of the topics under the broader research project on the SRH of 1.5-generation Bangladeshi women. The decision to end the first interview session was based on the participants’ availability. The length of the interviews per participant ranged from two to three hours. The sample size in this study was based on the depth of the data collected and coverage of the topics pertaining to the research questions. Small sample sizes in narrative inquiry are common because the richness of the data is emphasized (Kim, 2016; Riessman, 2008). Depth and richness of the data were achieved through multiple rounds of interviews with participants, exploring the social and cultural contexts of their lives, use of probing questions to delve deeper into topics relevant to the objectives of this study, re-reading interview transcripts, and reflexive engagement throughout the research process. The aim of this study was to highlight the stories shared by 1.5-generation Bangladeshi women and their unique experiences and perspectives on SRH-related informal and formal help-seeking. This study did not seek to generalize the experiences and perspectives of study participants and, therefore, data saturation was not an expectation, consistent with past studies that have employed the narrative inquiry methodology (Happel-Parkins & Azim, 2016; Kear, 2012; Whittam et al., 2021). The first author is a Bangladeshi-origin woman who grew up in the Middle East as a 1.5-generation migrant. Her cross-cultural positionality and similar ethnic and religious background helped foster rapport and trust with the participants, and provided important insight into the latter's SRH-related help-seeking behaviors and perspectives.

An interview guide was used to ensure coverage of relevant topics and maintain structure and consistency. Pre-determined, spontaneous and follow-up questions and conversations helped elicit narratives from participants. Since this study is part of a larger doctoral research project, data was collected on a wide range of topics, including participants’ childhood and upbringing, migration history, resettlement, knowledge about SRH, sex education, dating practices, and formal and informal help-seeking behaviors. In this paper, only the data related to SRH service access and utilization has been reported and other findings will be reported elsewhere (Nowshin et al., 2025).

Levesque et al.'s (2013) healthcare access framework guided data collection. Participants’ knowledge about SRH screening tests was assessed. They were asked whether they experienced SRH problems in their lifetime, what actions they took to address any SRH issues, who they sought help from regarding their concerns, what their service-seeking experiences were like, among other topics. The topics covered were related to the dimensions of the framework. For example, knowledge about SRH screening tests is linked with the demand-side dimension of “ability to perceive” of Levesque's framework, and sociodemographic preferences for physicians corresponds to the supply-side dimension of “acceptability”. An open stance was maintained to allow respondents to share SRH care or help-seeking experiences which may not be represented in the framework. Consistency of the data was ensured by asking participants to re-narrate some of their experiences and perspectives in follow-up interview sessions (Patton, 1999).

The interviews were transcribed verbatim by a professional transcriptionist and the transcripts were cross-checked with the audio recordings. The interview transcripts were coded using a qualitative data analysis software, QDA Miner Lite. Braun and Clarke's (2006) schema for thematic analysis was followed, which entailed familiarization with the data, generating codes, and identifying, reviewing, and defining the themes. Brief notes taken during data collection were also consulted. A combination of deductive and inductive approaches was used, and the coding framework (categories and codes that belong to each category) and thematic analysis were carried out iteratively, with new important codes and themes being included once identified. Data were analyzed to check whether and how they fit into the dimensions of Levesque's framework. For example, the codes “ethnicity preference for physicians” and “gender preference for physicians” led to the theme of embeddedness of culture in the SRH care-seeking process which corresponds to the dimension of “acceptability” of Levesque's framework. Identifying themes by analyzing different cases is an established practice in qualitative research (Riessman, 2008). By identifying the common elements across cases, it was possible to understand, for example, what the most prominent barriers to SRH care are for 1.5-generation Bangladeshi women. However, it was also important to highlight the less common, nuanced perspectives and experiences of study participants and throughout the process of data analysis, careful attention was paid to these narratives as well.

The first author conducted the data analysis. The usage of a single coder in qualitative research increases the risk of bias. To reduce these risks, the first author employed reflexivity during data analysis (evaluating the researcher's positionality, preconceived notions and assumptions), read the interview transcripts multiple times for strong familiarization with the data, and reviewed the coding framework.

## Results

The findings have been organized into four broad sections guided by the themes that emerged from the data: (1) Barriers to accessing SRH care; (2) Facilitators of access to SRH care; (3) Physician preferences; and (4) Informal SRH-related help-seeking behaviors. The themes and the corresponding sub-themes have been outlined in Table 2.

**Table 2.** The Major Themes and Corresponding sub-Themes That Emerged from the Data.

Themes	Sub-themes
1. Barriers to accessing SRH care *The barriers to accessing SRH care can be categorized into five sub-themes, which are detailed in the column on the right*	1.1 Limited knowledge about screening tests and available SRH services *Limited knowledge about names of routine SRH screening tests for women, what the tests screen for, alternative ways to access the tests beyond the family doctor* 1.2 Lack of proactiveness among primary health providers *Treatment plans only working in the short term, lack of comprehensive information about the different types of available ultrasounds, lack of proactiveness in the management of SRH issues which led to delayed diagnoses of PCOS* 1.3 Concerns about privacy and confidentiality *Fear of family members finding out about participants accessing SRH services through the family doctor, lack of trust in family doctors* 1.4 Pandemic-driven shift to virtual care *Patient-doctor communication gap driven by the virtual nature of care compounded by short duration of virtual consultations* 1.5 Insufficient female health providers *For some SRH services, appointments with female health providers can result in longer wait times*
2. Facilitators of access to SRH care *The facilitators of access to SRH care can be categorized into three sub-themes, which are detailed in the column on the right*	2.1 Higher education *The transition to university resulted in increased options in health facilities such as university clinics* 2.2 Established relationships with doctors *The long duration of acquaintance with the family doctor instilled trust and eased access to health services* 2.3 Enforcement of confidentiality policy of clinics *Trusting the health facility/providers to enforce the doctor-client confidentiality policy*
3. Physician preferences *Physician preferences depend on the patient's social, cultural, and religious values and beliefs as well as past experiences in health settings*	N/A
4. Informal SRH-related help-seeking behaviors *Mothers, peers, the internet, and mobile apps provided informal support on SRH issues, especially menstruation related problems.*	N/A

### Barriers to Accessing SRH Care

In this section, the barriers to accessing SRH services have been organized into five sub-themes: (i) Limited knowledge about screening tests and available SRH services, (ii) Lack of proactiveness among primary health providers, (iii) Concerns about privacy and confidentiality, (iv) Pandemic-driven shift to virtual care, and (v) Insufficient female health providers.

#### Limited Knowledge About Screening Tests and Available SRH Services

Respondents’ knowledge about routine SRH-related screening tests for women was explored. When asked to name these tests, nearly all respondents referred only to the Pap test. Some did not know what the test screens for and the age at which women are advised to take the test. Three respondents mentioned that the Pap test is recommended for women who are sexually active. Participants could not name other routine screening tests. One respondent who was unable to name any SRH-related screening test for women stated that she did not know how to access the tests:

“I don’t think I have done any of them. Well, honestly, I'm not really sure, like, what I would need to do the tests. But I know we should, right?” (P_10)

Two participants, aged 21 and 22, stated that their family doctor never discussed Pap tests or inquired whether they are sexually active, respectively.

“She [family doctor] never gave me a Pap smear ever. It's also important if you’re sexually active, which she never even asked.” (P_5)

Several respondents stated that they plan on taking the Pap test. Only one young woman who was sexually active underwent STD/STI screening. Overall, utilization of SRH screening tests among participants was low.

All participants were covered under the Ontario Health Insurance Plan (OHIP). The family doctor was the first and primary point of contact in formal help-seeking for SRH issues. Primary physicians play a pivotal role in administering SRH care and communicating pertinent information, which could affect patients’ ability to access essential SRH services. There exists a lack of knowledge about avenues for accessing such services beyond the family doctor, as illustrated by the excerpt below:

“I don't even really know what services would be available. My first point of contact for accessing these services would probably be my family doctor. I'm not sure if there's a central website or something that exists where I can get a list of services that are available to me. There definitely should be one, I think. Other than through my family doctor, I'm not really sure what services are even available.” (P_2)

#### Lack of Proactiveness among Primary Health Providers

Participants expressed dissatisfaction with their experiences of availing SRH care from their primary health providers. They felt that their concerns and queries were not being heard or addressed properly by their family doctors. Their anecdotes underscored dissatisfaction with treatment plans because they only worked in the short term, a lack of information about the different types of ultrasounds that are available, and a lack of proactiveness in the management of SRH issues which led to delayed diagnoses of polycystic ovary syndrome (PCOS).

Two women who began experiencing irregular, painful menstruation from 10 to 13 years of age were diagnosed with PCOS much later. In one case, painful menstrual cramps led to emergency care and intervention. The other respondent mentioned that prescribed medications did not alleviate discomfort from prolonged irregular, painful menstruation and pointed out the lack of comprehensive information about ultrasounds from her family doctor:

“My periods have never been regular, and they have always been really, really painful. I went to the family doctor when I was 10/11. But I was kind of dismissed because she was like, ‘It's just because you’re young, once you have your periods for a few years, it will change, you’ll be fine.’ So, then I waited a few years and my periods never really got normal. They continued to be really painful and I have a heavy flow … I kept going to my doctor and I kept on telling her that every time I went. And then finally she gave me an ultrasound and a blood test. And it was found that I had a large cyst, and then I had PCOS and just bunch of all those things, fibroids. That was in 2019, so I was 17 at the time, and that's when she prescribed me birth control and a couple of other medication that I was on … I took birth control all year throughout until last year when I just noticed that it wasn’t really helping anymore … This was last year and by this time I had a new doctor. She was the one who asked me to get a vaginal ultrasound done, which, by the way, I didn’t even know a vaginal ultrasound was a thing. She explained to me that a normal ultrasound from the outside, you can’t even see the cysts properly. So, it was an interesting thing because my family doctor never talked to me about a vaginal ultrasound. I had only been getting the regular ultrasound.” (P_6)

Another reason for dissatisfaction with the SRH care received from primary health care providers was insufficient information about prescribed medications. One interviewee who was prescribed birth control by her family doctor visited a private clinic where the doctor noted that the patient has a history of migraine. The doctor at the clinic mentioned that patients with a history of migraine should be cautious about using birth control because it can increase certain health risks. Upon hearing this advice, the participant felt that this is something that her family doctor should have brought up. She shared:

“The doctor [at the clinic] said, ‘You don’t prescribe birth control to patients who have migraine because a common side effect of being on any birth control is developing a blood clot which can travel to your brain and trigger an aneurysm or stroke.’ She explained that people with migraine are already at risk and taking birth control only increases that risk. So, I feel like this is something that my family doctor should have told me. He knew about my history of migraine.” (P_3)

#### Concerns About Privacy and Confidentiality

Breach of confidentiality emerged as another common concern among participants in this study. The family doctor is usually well-acquainted with parents of young patients because they are the primary health care provider for the family unit. Interviewees spoke about the risk of family members finding out about them accessing SRH services through their family doctor. Underlying their concern is a lack of trust in their doctors who they believe would reveal confidential information to their parents. One young woman spoke about the risks associated with seeking care for sensitive issues such as unintended pregnancy and abortion from the family doctor:

“If you're in high school, the family doctor would not be where you would go. That's the last place where you would go … because you cannot risk your parents or family knowing. So, it would be like trying to find those centers where they do abortions … But just not your family doctor.” (P_4)

Due to the cultural stigma associated with premarital sexual activity, unmarried, young Bangladeshi women may find it difficult to access essential SRH services. One married participant highlighted the issue of privacy for unmarried women accessing contraception:

“I feel like at this point I wouldn’t have anxieties about going to a doctor and asking about these services. But if I was two years younger than I am now, then I feel like I would definitely withhold because I was not married then, so contraception at that point … why would I need it? I could be judged, maybe, culturally, religiously, like if anyone saw me accessing these services when I’m not married. I feel like I would have had anxiety then.” (P_2)

#### Pandemic-driven Shift to Virtual Care

The shift to virtual appointments due to the Covid-19 pandemic contributed to a communication divide between family doctors and patients seeking SRH services. The lack of face-to-face interaction hindered patients’ ability to communicate their concerns effectively. The problem was compounded by the duration of virtual consultations which tends to be shorter than in-person appointments. Although this problem is not specific to the study population, it impacted one participant's SRH service-seeking experience. She spoke in detail about her unsatisfactory experience of availing virtual consultation with her family doctor. In addition to discussing birth control options, she sought a referral to a gynecologist to have a detailed discussion about her SRH given her family history of cervical cancer:

“It was near the end of last year, 2022. Even then my doctor wasn’t seeing patients in the clinic. He was still taking online and phone appointments. So I didn’t get to see him face to face. And my doctor has a lot of patients for a day. Again, the phone appointments with him are very rushed … even with my parents, anytime he's calling them or any of us, we’re lucky if the call lasts five minutes. It's usually like a three-minute call. We’ll blurt out what we need to, and he just gives a quick response, that's it. I definitely think that not having an in-person appointment contributed to it. Because it happened all so quick. I asked for a referral and he said, ‘Oh you don’t need a referral, I’ll write you a prescription.’” (P_3)

#### Insufficient Female Health Providers

One participant spoke about her experience of unsuccessfully trying to obtain SRH services delivered by female health providers. She stated that obtaining appointments with female technicians could result in longer wait times. She mentioned that compared to their male counterparts, female health providers are more understanding of women's SRH problems. According to her, men can be dismissive when women complain about menstrual cramps, pain or discomfort. This participant was trying to secure an appointment for a vaginal ultrasound. The following excerpt illustrates the difficulty she faced in obtaining an appointment with a female technician:

“I'm supposed to get a vaginal ultrasound, and I've been trying to book an appointment, and all the times that I keep getting are two/three months away and with a male technician which I'm not comfortable with. If I want a female technician, I have to wait much, much longer to actually get an appointment. And you know, I've been calling multiple clinics and they don't really have many female technicians working, which I feel like … it should not be this difficult for me to get an ultrasound.” (P_6)

### Facilitators of Access to SRH Care

The facilitators of access to SRH services have been organized into the following three sub-themes: (i) Higher education, (ii) Established relationships with doctors, and (iii) Enforcement of confidentiality policy of clinics.

#### Higher Education

As the young women in this study began higher education, they had the additional option to seek health services from university clinics. Earlier, their first point of contact was limited to the family doctor. However, the transition to university increased the array of health facilities to choose from. When asked whether she would go to the family doctor for SRH issues, one participant stated:

“I might go to the clinic in my university first, just because, like, I don't really know how to get in touch with him [family doctor] … That's of course if I'm in [university town]. But otherwise, I would tell my mom to make an appointment for me or something. It depends on the issue. I don't really know. Like, usually, when I talk to my parents about like, a health issue, or just like any issue that I need fixing … it takes a really long time for them to do it. Last year, I had that problem and then it took my parents like a year to make an appointment with an optometrist. So, it's usually just faster to do things myself.” (P_8)

#### Established Relationships with Doctors

Some participants stated that their family doctors were introduced to their parents through relatives and acquaintances. Many in the community availed services from the same doctor. Participants had known their family doctors for a long time, which fostered a sense of trust. One respondent harbored reservations about disclosing her SRH problems to her family doctor due to the perceived conservative background of the doctor. However, her mother's positive perceptions and experiences with the doctor alleviated her anxieties. Thus, the family doctor's link with immediate family members instilled trust:

“At the end of the day, this doctor has known me for a long time and if my parents can trust her … Because my mom went for her own sexual health issues. So, if my mom felt comfortable with it, and my family's okay with it, then I don't see why not.” (P_10)

Moreover, friendly relations between doctors and participants’ families helped ease access to health services. Although long wait times at family and university clinics and hospitals (to secure appointments or to see the doctor during visits) were reported by four participants, one young woman revealed that she was able to avoid long wait times because her family shares a friendly, informal relationship with the family doctor:

“I feel like the waiting times with my family doctor have been good, especially since like, we got to know these people. So, they kind of make exceptions for us and see us when we're in the clinic. We have his direct phone number and he calls just like normally, not as a doctor.” (P_8)

#### Enforcement of Confidentiality Policy of Clinics

Given the concerns about a breach of confidentiality and privacy when accessing services through the family doctor, the availability of alternative health facilities that ensure confidentiality enabled participants to access SRH services. Although the policy of confidentiality might be offered in the clinics that participants frequent with their families, participants did not necessarily believe that doctor-patient confidentiality would be protected in their family clinics. Other health facilities that routinely provide confidential SRH services and prioritize client confidentiality facilitated access to these services. Therefore, it is patients’ *trust* that the confidentiality policy will be enforced that is a facilitator of access to SRH services. One participant, who had embarked on a new relationship, was keen on taking an STI/STD screening test. She searched online to locate a private clinic that offered these services:

“I basically Googled ‘confidential STI testing Toronto’. And there was a list that came up from Toronto.health.ca or something. A list of various clinics. And I basically found the one closest to me and I called them and asked them about the services they offer and I made an appointment with them. It was pretty straightforward actually. But I think it was just like the fear of ‘Oh gosh, what if someone finds out?’ that was holding me back.’” (P_3)

### Physician Preferences

Participants were asked about their sociodemographic preferences for physicians when seeking SRH services. The findings show that physician preferences are shaped by a diverse set of social, cultural, and religious values and beliefs as well as past experiences in health settings. Demand for female physicians was voiced by the majority of participants while preferences with respect to ethnicity, age and religion of physicians were varied. Even among those who expressed a preference for female doctors, differences were observed. For example, discussing SRH problems with a male provider was deemed unacceptable by one respondent while others stated that they would opt for a female health provider only in cases where physical contact is required, pointing to varying degrees of modesty and religiosity among participants. Physicians of younger age were preferred because they are thought to be less judgmental and have better familiarity with Ontario's health system:

“I feel like a lot of the older doctors, they had their education back home, right? So, like a lot of the stuff definitely gets missed, when talking about this kind of stuff. I feel like the young doctors are more up to date with the system here compared to, like, the older doctors.” (P_9)

In terms of race/ethnicity and religion of physicians, preferences were mixed. However, participants’ stated preferences all reflect the cultural dimension of SRH care-seeking. One respondent stated that physicians of older age with a religious background may harbor cultural attitudes that may make it difficult to have discussions about SRH problems. Two participants exhibited a preference for non-white doctors for different reasons. One of these respondents felt that non-white doctors would understand the cultural context of navigating the SRH care-seeking process given the involvement of the family, reflecting the collectivist values of Bangladeshi culture. The other respondent took a more individualistic perspective and had a narrower set of sociodemographic criteria for her preferred physician. According to this participant, female doctors with a similar ethnic and religious background as her would be able to relate to her SRH problems:

“I think, I would love for my doctors to be as close as possible to what my description would meet. So, like a Muslim female *hijabi* doctor, Muslim female brown doctor, you know? Somebody who has the credentials, like obviously, they're doctors, but like somebody who can speak to my experience or what they've seen, even though it might be confidential, but what they've seen of women who are like me in my predicament.” (P_7)

### Informal SRH-related Help-seeking Behaviors

Mothers, peers, the internet, and mobile apps were sources of informal help-seeking. Respondents obtained SRH advice and information on irregular menstruation, menstrual cramps and cycles, and menstrual hygiene management.

Family members, particularly mothers and sisters, played an important role in conveying information regarding SRH problems which sometimes influenced formal help-seeking behaviors. SRH-related advice often involved irregular menstruation which was found to be a common issue among participants. Those who shared their SRH concerns with their siblings always turned to their older sister who had already experienced similar SRH problems. However, mothers were the most significant authoritative figure who influenced some participants’ perceptions about SRH problems and decision-making regarding whether to seek medical care. The following incident illustrates the persuasive power that mothers wield in participants’ decisions to seek formal help:

“I’ve talked to my mom a couple times pushing her, because I heard, birth control is something a lot of people use to make their periods regular. And then she was just like, ‘No, you don’t need to do that cause of side effects. It's not going to be good for you.’ And so, we haven't even bothered to ask the doctor about it, because my mom has been educated on a lot of things about health in general, so she was like, ‘It's going to help but unless it was very serious, you don't need to do birth control just because the side effects are way too much.’” (P_4)

Not all participants complied with the advice received from their mothers. Two respondents, who were determined to seek medical help, expressly disagreed with the advice they received from their mothers regarding management of menstrual cramps and irregular periods. One of them was keen on using birth control pills to ease menstrual cramps, which her friends had done successfully. Her experience reflected the tensions that can arise as a result of conflicting information received from peers and mothers. The other respondent did not believe her family members understood the underlying cause of her SRH issue. Negative experiences with family members related to past health issues and a perceived lack of social support triggered a lack of confidence in them. She shared her parents’ reaction to her experience with irregular menstruation:

“I’ve always had really irregular periods and that got dragged into this as well, like ‘Oh, it's because you’re gaining so much weight.’ And this during COVID when I was depressed and then obviously, they didn’t notice that either. And that comes up, and they make a really big deal out of that stuff, like ‘Oh, this is not good. It's not good to have irregular periods.’ Obviously, they’re concerned about fertility, but they don’t come out and say that. They just make it your fault like, ‘You’re not taking care of yourself, that's why this is happening.’” (P_5)

Eventually, both women sought consultation with their family doctors regarding their SRH problems. Relative to other participants in this study, they exhibited increased personal autonomy with respect to SRH care decision-making by going against parental advice.

Peers were consulted to a lesser degree compared to mothers. Participants were keen on learning about their friends’ SRH problems while simultaneously discussing their own SRH concerns in the hope of alleviating anxieties and garnering support. A sense of comfort in peer-to-peer relationships prompted such conversations. An additional factor could be participants’ perception that their SRH issues were contemporaneous with those of their peers.

“I feel like when I get my periods and I'm really like hurting, the whole world will know. Like, I’ll even tell my dad, you know? If I suffer, the whole world suffers with me. But I think my friends and I, we sort of have that camaraderie, where it's like ‘Okay, you tell me what's going on. I'll tell you what's going wrong, and we'll get through it together’ type of thing.” (P_10)

Peers were also found to have knowledge about modern products related to menstrual hygiene management. One respondent was persuaded by her friend to start using menstrual cups which she found to be eco-friendly and more convenient to use than sanitary pads.

The internet and mobile apps emerged as other sources of informal help-seeking. One participant stated that after being diagnosed with PCOS and endometriosis, she resorted to the internet to gather more information about these issues. The internet was used as a supplemental support to formal help-seeking. Another participant whose menstruation was irregular mentioned using a mobile app to track her menstrual cycle upon being persuaded by her friend. She stated:

“I downloaded an app to make sure I track it [period] after my friend was like, ‘You should just track your period so you can expect it. It definitely doesn't come out of the blue.’ You can always like log in and see what the flow was like, how the pain was, and it helps with predicting your next cycle.” (P_4)

## Applying Levesque's Framework

**Figure 2.**
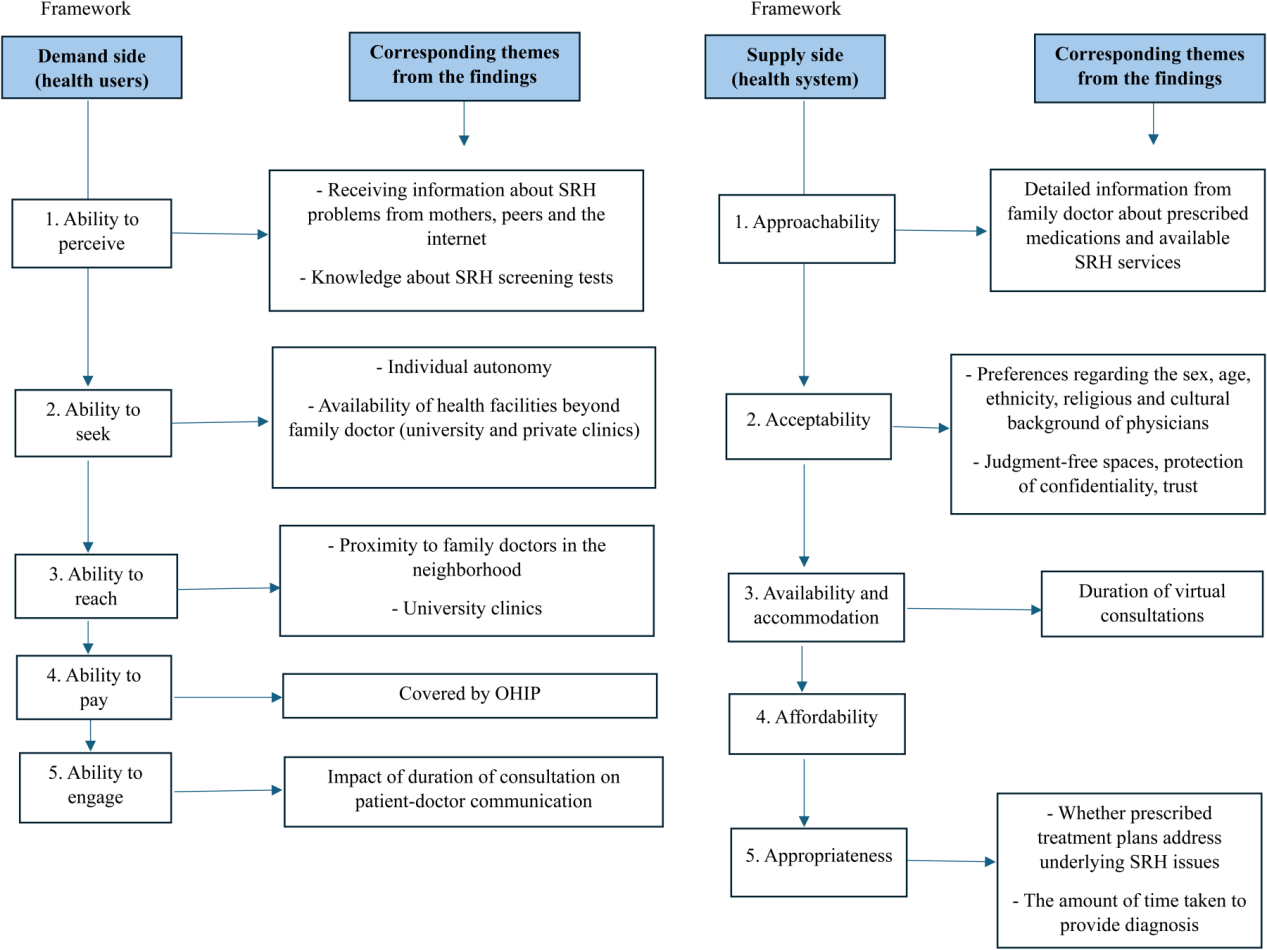
Mapping the Findings Across the Demand- and Supply-side Dimensions of the Conceptual Framework of Access to Healthcare Developed by Levesque et al. (2013).

The findings were mapped along the sequential demand- and supply-side dimensions of the framework by Levesque et al. (2013). Figure 2 illustrates the factors that emerged as significant in the SRH care trajectories of 1.5-generation Bangladeshi women. The framework highlights the linkages *along* the dimensions on the demand and supply sides (e.g., “ability to perceive” and “ability to seek”) as well as *between* the corresponding dimensions (e.g., “ability to perceive” and “approachability”) in SRH service access and use. Relevant factors in any of these dimensions can either disrupt or facilitate access to SRH care. For example, limited knowledge about the recommended timing and purpose of Pap smears (“ability to perceive”) prevents participants from seeking cervical screening services (“ability to seek”), and the SRH care trajectory is disrupted due to insufficient knowledge about Pap smears. While there are factors that act as facilitators (e.g., higher education, OHIP coverage), barriers to SRH care were observed at different stages of both the demand- and supply-side dimensions.

## Discussion

This study explored the SRH-related help-seeking behaviors and experiences and perspectives of accessing and utilizing SRH services among young 1.5-generation Bangladeshi women in Toronto. The findings shed light on the barriers to SRH care, including limited knowledge about screening tests and available SRH services, a lack of proactiveness among primary health providers, and concerns about privacy and confidentiality. The facilitators of access to SRH care were higher education, established relationships with doctors, and enforcement of confidentiality policies of clinics. Informal sources of support were mothers, peers, the internet, and mobile apps.

Knowledge about SRH screening tests and utilization of these services were found to be low. Knowledge about the importance of HPV, Hepatitis C, and gonorrhea screening was lacking. Participants did not mention the importance of routine screening for these STIs (CDC, 2024). Low levels of knowledge about the availability and purpose of screening tests partly stemmed from limited discussions about SRH with family doctors.

Differences were observed in the nature of help-seeking from mothers versus peers. While mothers provided advice and instructions, peers were a source of learning about mutual experiences, reflecting the hierarchical dynamics of mother-daughter relationships. In some cases, mothers played an important role in shaping perceptions about medical needs and decisions to seek care with respect to SRH. SRH issues discussed with mothers were restricted to irregular menstruation. Topics directly related to sexual practices were avoided, consistent with previous research that has highlighted the lack of parent-child communication about sex-related topics among South Asian adolescents in Canada (Meherali et al., 2021). The literature on the role of social support predicting formal help-seeking behaviors is varied (Brown et al., 2014; Grewal et al., 2005; Hedge et al., 2017; Rickwood & Braithwaite, 1994; Saunders et al., 1994). In this study, the differing perceptions of SRH problems between some participants and their mothers and the lack of social support from family did not necessarily inhibit professional care-seeking. This echoes the findings of Dune et al. (2017) who reported that 1.5-generation migrants in Australia did not believe that their SRH help-seeking behaviors were shaped by the perceptions of others. The internet/technology was used in combination with formal and informal help-seeking. Usage of online sources as an adjunct to professional help-seeking is encouraging and is consistent with findings of other studies (Collin et al., 2011; Gould et al., 2002).

Individual-level social and cultural factors were apparent in participants’ help- and care-seeking attitudes. For example, seeking help from mothers and valuing their opinions reflect the collectivist ethnocultural values of 1.5-generation Bangladeshi women (Dune et al., 2021). A preference for female health providers for medical examinations that entail close physical contact (e.g., vaginal ultrasound) points to participants’ values of modesty. Availability of female health providers is, therefore, an important consideration in the formal help-seeking intentions of 1.5-generation Bangladeshi women. Health providers who understand the cultural beliefs and values associated with SRH can improve the service experience, for example, by approaching the care-seeking process with sensitivity if family members are involved.

Structural factors in the health system context hindered the effective delivery of SRH services, exemplified by the lack of proactiveness of primary physicians and the short duration of consultations. The former highlights the urgency to adopt a judicious, patient-centered approach in the SRH care of young women. The duration of consultations depends on factors such as physician burnout, shortage, and administrative burden which continue to plague Ontario's health system (Jones, 2023). Suboptimal quality of SRH care can discourage young women from seeking further medical attention (Taber et al., 2015). A patient-doctor communication gap was observed, stemming from the perceived time pressure of consultations which can disincentivize patients from asking questions (Beisecker & Beisecker, 1990; Pollock & Grime, 2002). Participants’ young age may have contributed to the power imbalance in the client-provider relationship and their limited capacity to communicate SRH issues effectively and participate fully in the care-seeking process. Trust, emotional safety, and autonomy are important features for young patients, and health providers need specific communication skills to engage with young clients (Chant et al., 2002; Kim & White, 2018).

The family doctor is the most significant source of formal help-seeking and, therefore, plays a critical role in providing primary SRH care and facilitating access to specialized services. Participants did not receive adequate information from their family doctors about prescribed medications and available SRH services. The provision of comprehensive, accurate information by family doctors is essential to improve access to SRH services and ensure patient satisfaction (Al-Ahmadi & Roland, 2005). Participants expressed concerns about breach of confidentiality and privacy when visiting the family doctor for their health problems. These concerns were heightened for accessing care for issues such as abortion and contraception due to the likelihood of family and community members finding out and the stigma related to premarital sexual activity. These barriers have been reported in previous research on South Asian immigrant adolescents in Canada (Meherali et al., 2021). Such concerns may lead young people to refuse routine screening tests and follow-up care (Ford et al., 1997). Health providers should promote the privacy and confidentiality of SRH services and aim to foster a sense of trust among young patients so that the latter feel safe in accessing SRH care. Trust in physicians encourages disclosure of health information and facilitates timely diagnoses (Calnan & Rowe, 2006). Young patients’ autonomy and informed consent and non-judgmental attitudes of health providers are integral components of youth-friendly SRH services (UNFPA, 2021). Health promotion efforts are recommended in Toronto neighborhoods with a predominantly Bangladeshi population (e.g., Danforth). SRH programming in these neighborhoods should focus on imparting awareness about routine SRH screening tests and other types of SRH care, and available youth-friendly SRH services.

Importantly, the findings reflect the role of acculturation in the SRH care-seeking attitudes of the 1.5-generation women in this study who immigrated to Canada at a young age. Acculturation can be broadly defined as “the dual process of cultural and psychological change that takes place as a result of contact between two or more cultural groups and their individual members” (Berry, 2005, p. 698). While there exist cultural dimensions to the SRH care-seeking process, participants did not exhibit reluctance to speak to their doctors, suggesting an openness to talk about SRH problems likely due to their exposure to formal SRH education and acculturation to Canadian society. Similarly, Luu et al. (2020) found that 1.5- and second-generation Vietnamese-American women were willing to talk to their primary care providers about their gynecological concerns. However, older migrants who were socialized in their country of origin and are less acculturated to the host society may feel hesitant to speak about their sexual health issues with their doctors due to feelings of shame and embarrassment (Ilami & Winter, 2021; Maticka-Tyndale et al., 2007; Metusela et al., 2017). In their study on the SRH help-seeking attitudes of 1.5-generation migrants in Australia, Dune et al. (2021) observed that “1.5 generation migrants are not influenced by culture to the same extent as their older counterparts” (p.9). The effects of acculturation are also implicit in participants’ adherence to Western medicine when it comes to SRH, fluency in English, and familiarity with the Canadian health system. On the other hand, the need for culturally specific health services, language barriers, and difficulty navigating the host country's health system are common challenges faced by newcomers and older migrants when accessing health care (Dastjerdi et al., 2012; Lai & Chau, 2007; Maticka-Tyndale et al., 2007; Meherali et al., 2021; Wang et al., 2008). The findings reflect the importance of considering generational status in the analyses of help-seeking attitudes of immigrant populations, consistent with previous research that has identified generational cohort as an indicator of the level of acculturation and health care utilization patterns (Glaesmer et al., 2011; Rumbaut, 2004).

Culturally competent care can reduce health inequities and improve the efficiency of resources by reducing medical errors (Anderson et al., 2003; Butler et al., 2016). However, there is limited understanding of how cultural competency can be implemented effectively on the ground (Carpenter-Song et al., 2007). Critiques of cultural competency include risks of overestimating cultural differences and conflating culture with race or ethnicity (Carpenter-Song et al., 2007). These limitations are relevant to the findings of this study which showed that acculturation shaped the SRH-related help-seeking behaviors of 1.5-generation Bangladeshi women in different ways although their ethnic background is the same. For example, only one participant was unwilling to discuss SRH problems with a male health provider. Others did not exhibit an unwillingness to discuss their SRH issues based on the sex of the health provider. Further investigation is required to better understand what culturally responsive SRH care for 1.5-generation patients from South Asian and Muslim backgrounds entails. Clinicians’ perspectives can shed light on the challenges they face in delivering SRH care to young South Asian patients belonging to different generations and cultural/religious backgrounds.

This study has some limitations. This was a qualitative study with a limited purposive sample. Thus, the findings cannot be generalized to other contexts. Participants’ experiences with accessing SRH services took place in an urban context. Therefore, their narratives may not reflect the experiences of similar populations living in rural or semi-rural areas where the barriers to SRH care may be heightened due to geographical and social isolation among other factors. Absent in this study are perspectives of actors within the health system (e.g., physicians, health services managers) which would have provided more insight into the supply-side dimensions of SRH care access.

## Conclusion

Knowledge about routine SRH screening tests and utilization of these services are low among young 1.5-generation Bangladeshi women in Toronto. The SRH needs, informal help-seeking behaviors, decisions to seek formal care, and perspectives and experiences with accessing SRH services were varied among participants. The interlinked demand- and supply-side dimensions of SRH care show how barriers in any of these dimensions can disrupt access to quality care. This paper underscores the importance of recognizing the heterogeneity of immigrant groups and considering culture and generational cohort in understanding their SRH-related care-seeking behaviors. A patient-centered approach is necessary in the SRH care of young 1.5-generation Bangladeshi women. Educational campaigns in residential neighborhoods concentrated by the Bangladeshi diaspora are recommended. Information about the importance and purpose of SRH care and available youth-friendly SRH services should be disseminated in the wider community, including clinics and schools. Further investigation is required to understand what culturally competent care entails for 1.5-generation patients from South Asian and Muslim backgrounds. This study adds to the limited, yet growing, body of research at the intersection of 1.5-generation and SRH. Future research should explore the SRH-related help-seeking behaviors among other immigrant youth populations.

## Notes

1. These services refer to the broad array of formal services that address sexual and reproductive health problems and needs (irregular menstruation, contraception, STD/STI testing, cervical screening, etc).
